# Accelerated germination of aged recalcitrant seeds by K^+^-rich bulk oxygen nanobubbles

**DOI:** 10.1038/s41598-023-30343-2

**Published:** 2023-02-27

**Authors:** Mijung Kim, Akio Shoji, Toshiaki Kobayashi, Yasuyuki Shirai, Shigetoshi Sugawa, Masayoshi Takahashi

**Affiliations:** 1 Special Technical Team, Oriental Shiraishi Corporation, 5-6-52 Toyosu, Koto-ku, Tokyo 135-0061 Japan; 2 Graduate School of Life and Environmental Sciences, University of Tsukuba, 1-1-1 Tennodai, Tsukuba, Ibaraki 305-8572 Japan; 3 New Industry Creation Hatchery Center, Tohoku University, Sendai, 980-8579 Japan

**Keywords:** Biological techniques, Biotechnology, Chemical biology, Plant sciences, Nanoscience and technology

## Abstract

Bulk nanobubbles, measuring less than 200 nm in water, have shown their salient properties in promoting growth in various species of plants and orthodox seeds, and as potential drug-delivery carriers in medicine. Studies of recalcitrant seeds have reported markedly increased germination rates with gibberellin treatment; however, neither the mechanism promoting germination nor the implication for food safety is well elucidated. In our study, recalcitrant wasabi (*Eutrema japonicum*) seeds treated with bulk oxygen nanobubbles (BONB) containing K^+^, Na^+^, and Cl^−^ (BONB-KNaCl) showed significantly accelerated germination. As germination progressed, 99% of K^+^ ions in the BONB-KNaCl medium were absorbed by the seeds, whereas Ca^2+^ ions were released. These results suggest that the germination mechanism involves the action of K^+^ channels for migration of K^+^ ions down their concentration gradient and Ca^2+^ pumps for the movement of Ca^2+^ ions, the first potential discovery in germination promotion in recalcitrant seeds using nutrient solutions with BONB-KNaCl.

## Introduction

Nanobubbles (NBs) are known to show various effects in plants and living organisms, including accelerated growth in bacteria, plants, and animals, increased germination rate in barley seeds, and targeted drug delivery of cancer therapies by exposure to continuously generated NB solution. However, the mechanisms of how these substances play such roles are not well understood^1–9^.

Recently, there has been an increase in literature studies of bulk nanobubbles (BNBs), which are tiny NBs in water measuring less than 200 nm^10,11^. BNBs have the unique characteristic of stability in water for prolonged periods even though the stabilization mechanism is still unclear^12^. Our previous study proved the existence of BNBs through physicochemical methods such as atomic force microscopy (AFM) and electron spin resonance (ESR) spectroscopy, which reported bubble sizes smaller than 50 nm^12^.

BNBs have been used in a variety of medical settings, ranging from drug delivery to ablation of tumours^9^, with most use on in vitro cultured cells. Despite the increased research attention on BNBs and their potential applications, there has been limited research on their fundamental mechanisms, which have scope for application in plant models. Given that plant seeds must be germinated for plant cultivation to proceed, the seed germination rate is a critical measure when examining the relative merits of seed treatments. Recalcitrant seeds are so described because they show poor germination rates after storage, which is typically caused by the high sensitivity of the seed to desiccation^13^. Although most seeds remain viable under dry storage conditions, recalcitrant seeds will not germinate after dry storage because they require specific conditions of moisture content and temperature. Therefore, use of old and/or recalcitrant seeds in an experimental program allows any enhancements in germination to be readily identified.

Wasabi (*Eutrema japonicum*), typical recalcitrant seeds, are notorious for their sensitivity to temperature, pH, dissolved oxygen level, and the chemical environment of the surrounding water, making it difficult to germinate even fresh seeds^14–17^. Depending on the cultivation setting for these seeds, they are designated as soil-wasabi or water-wasabi. Water-wasabi is a high-value agricultural product because of its fastidious germination and cultivation methods. The traditional method of germination of water-wasabi is to soak the seeds in the flowing water of mountain streams in specific regions of Japan at temperatures of 8–18 °C. This setting provides a constant supply of dissolved oxygen to the seed, increases the permeability of the seed coat, and facilitates gas exchange within the developing embryo^18–21^. For most plant crops, the germination rates of commercial seeds after drying and storage are over 80%, whereas the germination rate of wasabi seeds is zero after 2 weeks of storage^14,15^. With such low germination rates and difficulty in controlling the germination environment, farmers have adopted tissue culture for mass production of water-wasabi^22^. However, a disadvantage of tissue culture is the transformation of species from mutations, which can cause loss of species diversity. Tissue culture is also not suitable for developing new varieties of wasabi. Improving the germination rate of high-value water-wasabi is expected to increase farm income and strengthen industry competitiveness through the development of new varieties. In addition to water-wasabi, other valuable plant crops such as mango, lychee, jackfruit, oil palm, and cocoa, which may show price increases linked to supply problems caused by climate change, COVID-19, or the Ukraine conflict, can benefit from this technology. In other studies of wasabi germination, although the germination rate of recalcitrant wasabi seeds increased with gibberellin treatment, neither the mechanism promoting germination nor the implication for food safety is well elucidated^23^.

Through the functional roles of BNBs in enhancing the attraction of cations such as calcium, potassium, and sodium, gas bridging and transfer, and formation of reactive oxygen species (ROS), we considered that the germination process could be analysed to elucidate the steps involved. Several objectives were addressed in this study: (1) to investigate the mechanism of germination through BNB involvement; (2) to investigate the mechanism of stability of BNBs with ions; and (3) to confirm the potential of using BNBs with ions in difficult plant cultivation using aged recalcitrant seeds. We believe that production of ROS as BNBs diminish is the main stimulus for seed germination through a synergistic mechanism involving BNBs and cations.

## Results

### Accelerated germination by BONB water

**Figure 1:**
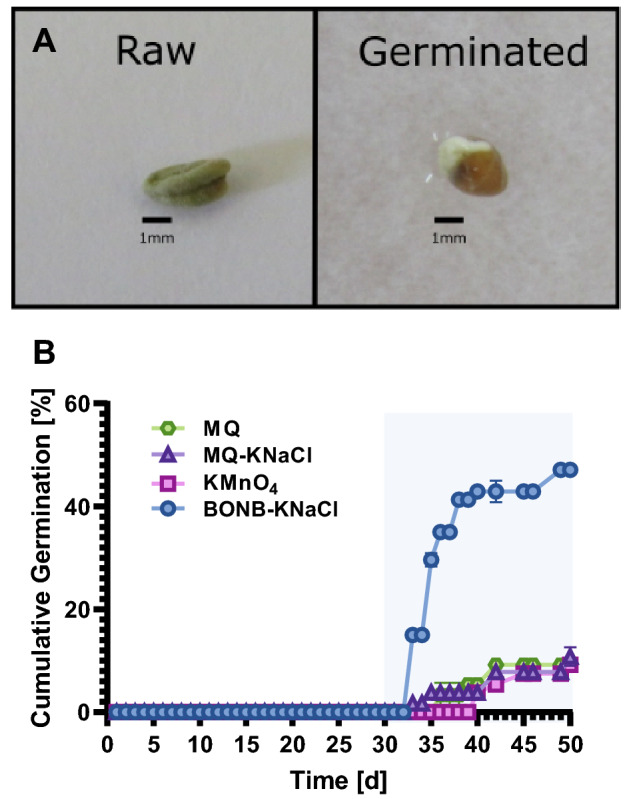
Average cumulative germinations of experiment 1. (**A**) Left image shows an example of a raw seed; right image shows a germinated seed. (**B**) Cumulative germination rates of wasabi seeds after conditioning in treatment solution: MQ (milli-Q water), MQ-KNaCl (milli-Q water containing K^+^, Na^+^, and Cl^−^), KMnO_4_ solution, and BONB-KNaCl (bulk oxygen nanobubble solution containing K^+^, Na^+^, and Cl^−^). Figure shows mean data from duplicate experiments. Error bars indicate standard errors. Data points without error bars indicate no standard error.

Our study looked at the relationship between bulk oxygen nanobubbles (BONB) and ions in solution in promoting germination of aged recalcitrant seeds of water-wasabi (Fig. 1A). To stabilize BNBs in the germination medium, we used sodium-based solutions and added several different ions to compare their effects on germination (Table 1).

**Table 1:** Properties of culture media used in the experiments.

Solution	Ion concentration				EC [mS/cm]	Initial pH	Initial DO [mg/L]
	Na^+^ [mg/L]	K^+^ [mg/L]	Ca^2+^ [mg/L]	Cl^−^ [mg/L]			
Experiment 1							
MQ (control)	0.00	0.00	0.00	0.00	0.00	5.73 ± 0.01	8.0 ± 0.0
MQ-KNaCl	147.11 ± 8.91	289.47 ± 0.05	0.00	566.22 ± 9.97	1.66 ± 0.00	6.47 ± 0.01	8.0 ± 0.0
KMnO_4_	145.55 ± 0.80	285.45 ± 3.51	0.00	561.63 ± 0.46	1.64 ± 0.00	6.49 ± 0.14	8.0 ± 0.0
BONB-KNaCl	145.58 ± 1.29	298.01 ± 0.47	0.00	565.40 ± 3.74	1.68 ± 0.00	6.77 ± 0.01	8.0 ± 0.0
Experiment 2							
MQ (control)	0.00	0.00	0.00	0.00	0.00	5.73 ± 0.01	8.0 ± 0.0
MQ-KNaCl	102.70 ± 0.40	292.49 ± 7.41	0.00	472.55 ± 0.17	1.68 ± 0.00	6.48 ± 0.01	8.0 ± 0.0
MQ-CaNaCl	104.14 ± 0.13	0.00	187.06 ± 0.10	472.92 ± 0.41	1.68 ± 0.00	6.56 ± 0.14	8.0 ± 0.0
BONB-KNaCl	103.24 ± 0.14	291.08 ± 9.90	0.00	472.68 ± 1.75	1.68 ± 0.00	6.82 ± 0.03	8.0 ± 0.0
BONB-CaNaCl	103.86 ± 0.24	0.00	186.70 ± 0.18	472.88 ± 0.54	1.68 ± 0.00	6.87 ± 0.06	8.0 ± 0.0

EC was measured at 15 °C. Values are presented as mean ± SE.

*EC* electrical conductivity, *DO* dissolved oxygen, *MQ* Milli-Q water, *MQ-KNaCl* Milli-Q water containing K^+^, Na^+^, and Cl^−^, *BONB-KNaCl* bulk oxygen nanobubble water containing K^+^, Na^+^, and Cl^−^, *MQ-CaNaCl* Milli-Q water containing Ca^2+^, Na^+^, and Cl^−^, *BONB-CaNaCl* bulk oxygen nanobubble water containing Ca^2+^, Na^+^, and Cl^−^.

To determine the effects of the different ions on germination, we used Milli-Q water alone (MQ), KMnO_4_, and solutions of MQ-KNaCl (K^+^, Na^+^, and Cl^−^ in MQ water) and BONB-KNaCl (K^+^, Na^+^, and Cl^−^ in BONB water). Significant improvement in germination rate was observed for seeds treated with BONB-KNaCl when compared with other germination solutions (Fig. 1B). The highest daily germination rate reached to 15.00% from day 33, and the cumulative germination was 47.08% on day 50. By comparison, although the germination increased gradually from day 33 (1.67%) for seeds treated with MQ-KNaCl and from day 40 (3.75%) for those treated with KMnO_4_, the 50-day cumulative germination of seeds treated with BONB-KNaCl was more than four times the rates of seeds treated with MQ-KNaCl (10.83%) or KMnO_4_ (9.17%).

We did not observe any effect of electrical conductivity (EC) on germination (Table 1). For BONB-KNaCl treatments across both experiments, we saw a range in Na^+^ concentration of 103.24–145.58 mg/L, but it was difficult to determine any difference between the germination rates from these two experiments based on Na^+^ concentration. The range of K^+^ concentration for all experiments featuring K^+^ was 285.45–298.01 mg/L. In each experiment, the highest cumulative germinations were for seeds treated with BONB-KNaCl, suggesting that there is a significant mechanistic effect involving K^+^ and BONB. Therefore, we considered that the concentration of K^+^ in solution was not as critical as the combined effect of K^+^ and BONB in water.

### Ionic effects with BONB water

Following our hypothesis that there is a synergistic effect between K^+^ and BONB, we compared the effects of BONB-CaNaCl and BONB-KNaCl on seed germination to discern the effects of the different ions. Transmembrane gradients of K^+^ and Ca^2+^ are known to be important for plant growth^24,25^. Previous studies have shown the importance of Na^+^/K^+^ ratio for plant growth and that H^+^-ATPase is one of the essential ion pumps that controls the concentration of K^+^ for plant growth^26^. However, the mechanism and the relationship between ion concentrations and seed germination have never been investigated. To discount the effect of BONB and its possible interaction with cations in germination, we also tested the effects of MQ-KNaCl and MQ-CaNaCl.

**Figure 2:**
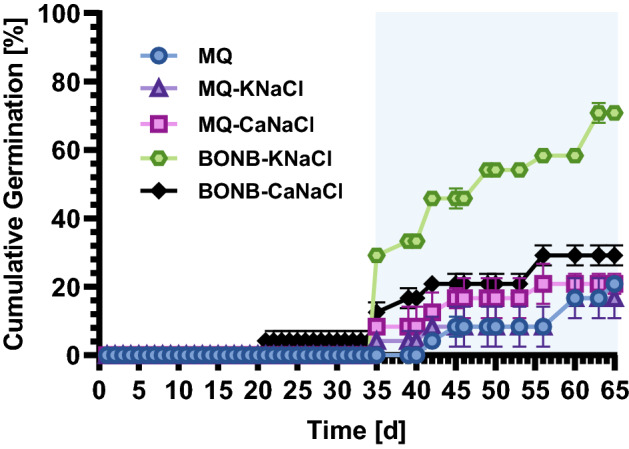
Average cumulative germinations of experiment 2. Figure shows cumulative germination rates of wasabi seeds after conditioning in treatment solution: MQ (milli-Q water), MQ-KNaCl (milli-Q water containing K^+^, Na^+^, and Cl^−^), MQ-CaNaCl (milli-Q water containing Ca^2+^, Na^+^, and Cl^−^), BONB-KNaCl (bulk oxygen nanobubble solution containing K^+^, Na^+^, and Cl^−^), and BONB-CaNaCl (bulk oxygen nanobubble solution containing Ca^2+^, Na^+^, and Cl^−^). Error bars indicate standard errors. Data points without error bars indicate no standard error.

When compared with MQ, ionic solutions promoted the germination of aged recalcitrant seeds. Although initial germination was first observed for seeds treated with BONB-CaNaCl, substantial improvement was not seen until day 35 for all the solutions except for the control (Fig. 2). On day 35, the cumulative germinations of seeds treated with BONB-KNaCl, BONB-CaNaCl, MQ-CaNaCl, and MQ-KNaCl solutions were in a decreasing order of 29.17%, 12.50%, 8.33%, and 4.17%, respectively. Germination for the control solution was not observed until day 42, starting at 4.17%. Surprisingly, the cumulative germination for MQ-KNaCl and MQ-CaNaCl did not show much improvement, suggesting that the ions themselves cannot promote germination. The BONB water solutions showed the strongest promotion of germination. BONB-KNaCl treatment showed the highest cumulative germination (70.83%) of aged recalcitrant seeds, which was 2.5 times higher than the germination of seeds treated with BONB-CaNaCl (Fig. 2). This result suggested a synergistic effect of BONB with potassium, sodium, and chloride ions.

### Uptake and releases of ions with BONB

**Figure 3:**
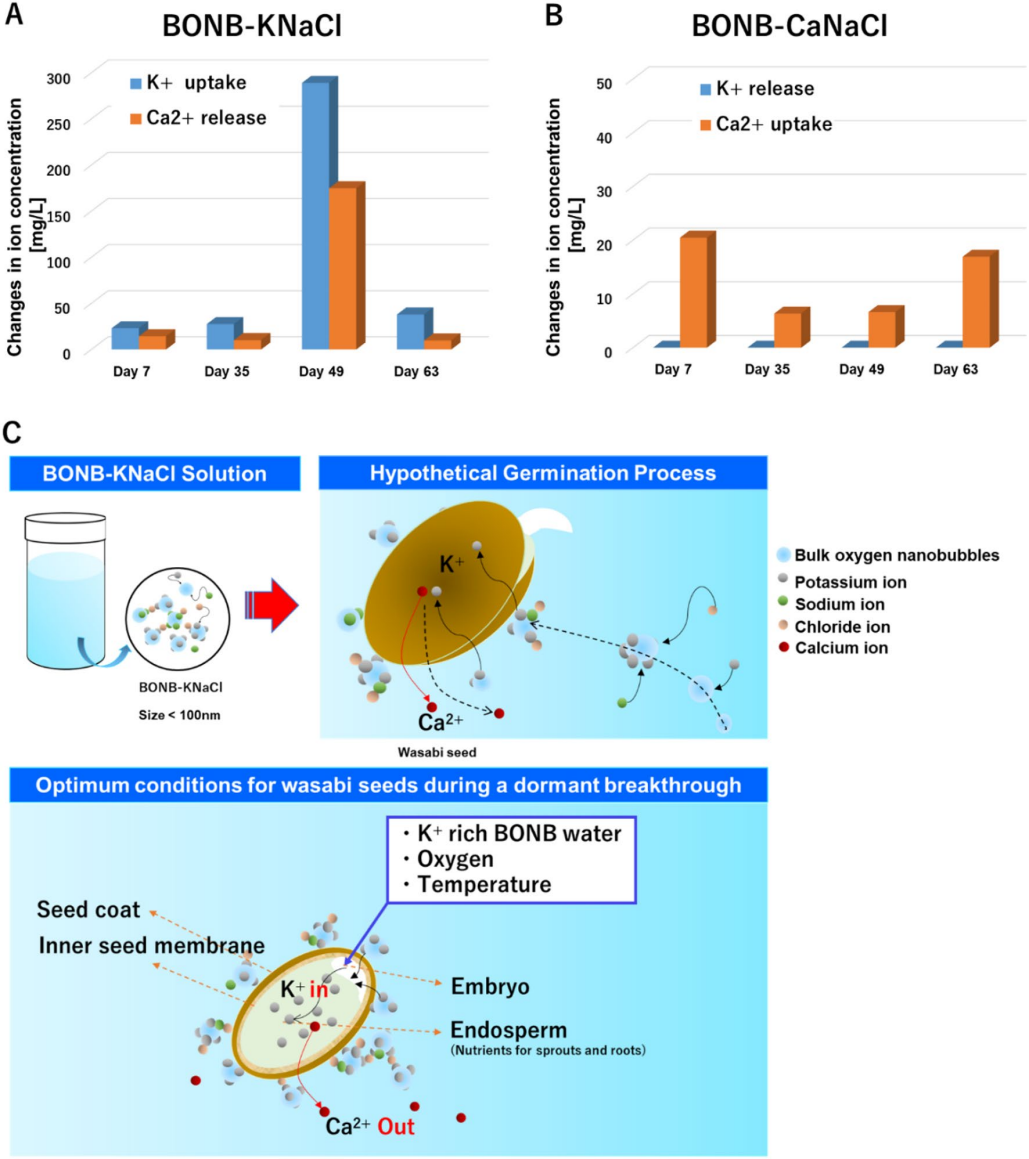
Uptake and release of K^+^ and Ca^2+^ ions. (**A**) K^+^ uptake and Ca^2+^ release from seeds in BONB-KNaCl. (**B**) K^+^ release and Ca^2+^ uptake from seeds in BONB-CaNaCl. (**C**) Proposed mechanism of K^+^ uptake and Ca^2+^ release in aged recalcitrant of wasabi seeds with BONB-KNaCl. Upper left shows an example of solution used in the study. Upper right shows a hypothetical model of the germination process. Lower panel shows optimum conditions for wasabi seeds to break out of dormancy, with significant K^+^ uptake from the solution and Ca^2+^ release from the endosperm.

In experiment 2, concentrations of the ion species in solution were determined by ion chromatography analysis. For the treatment of seeds with BONB-KNaCl, the absorption rate of K^+^ at day 49 of germination was 99.29% from an initial concentration of 291.08 mg/L. Over the monitoring period, the absorption rate of K^+^ from BONB-KNaCl was in a range of 7.93–99.29% (Fig. 3A). In contrast, the absorption rate of K^+^ from MQ-KNaCl stayed in the range of 7.94–10.46% throughout the germination period. For seeds treated with MQ-CaNaCl or BONB-CaNaCl, absorption of K^+^ was not detected, but the aged recalcitrant seeds in MQ released Ca^2+^ ions (0.13 mg/L or lower) throughout the germination period (data not shown).

For seeds treated with BONB-KNaCl, the Ca^2+^ ion concentration was 174.82 mg/L on day 49, suggesting that Ca^2+^ ions were drawn out by the synergistic effect of K^+^-rich BONB, possibly because of the toxic effect of Ca^2+^ ions on germination (Fig. 3A). In contrast, seeds treated with BONB-CaNaCl, with an initial Ca^2+^ concentration of 186.70 mg/L, showed Ca^2+^ uptake of 3.41–10.97% of the initial concentration but showed no release of K^+^ throughout the experiment (Fig. 3B).

All solutions showed release of Na^+^ throughout the germination period (data not shown). Seeds treated with BONB-KNaCl or BONB-CaNaCl showed low releases of Cl^−^ from day 35 to 49 (from 40.74 to 76.90 mg/L). Interestingly, from day 49 to 65, BONB-KNaCl seeds absorbed the most Cl^−^ (day 65: 74.56 mg/L), while BONB-CaNaCl seeds released Cl^−^ (day 65: 33.06 mg/L).

## Discussion

We investigated the relationship between ions and BONB in promoting the germination of recalcitrant wasabi seeds. To increase the stability of BONB in the medium, we used sodium-based solutions with several different ions that are reported to have massive effects on seed germination^27–29^ and compared their effects on germination. We measured the concentrations of ions in BONB solutions and observed significant changes in K^+^ and Ca^2+^ concentrations through passive and active movements of ions across the cellular membranes with help of ionic channels and proton pumps^30^. The control solutions containing K^+^, Na^+^, and Cl^−^ without BONB did not show any difference in total germination. Therefore, we not only report the facilitative features of BONB with electrochemical gradient changes but also emphasize the synergistic effect of BONB and cations in accelerating the germination.

Generally, the germination of seed is thought to occur through respiratory action. As the seed absorbs water via osmosis, the seed shell becomes loose, absorbing oxygen, while releasing CO_2_ in a gas exchange process. Concentrations of ions in the cells begin to increase, facilitating enzyme activity, activating hydrolysis of storage materials and other physiological actions, ultimately causing seeds to germinate. Our results suggest that for active movements of ions across the membranes, especially for treatment with BONB-KNaCl, BONB work as carriers of ions and provide an appropriate environment for ionic transportation because they are negatively charged, thereby attracting positively charged ions. It is likely that the ionic cloud surrounding the negatively charged BONB allows for internal protons to be released via proton pump, making the electrochemical gradient higher, and activating potassium channels to selectively receive potassium, which facilitates enzyme activation. Moreover, ROS, important signalling molecules for gibberellin, increase in concentration as BONB diminish, which helps to break the dormancy and enhance seed germination^31^.

As observed in Figs. 1B and 2, the number of germinated seeds increased soon after 30 days of treatment. From this point, we assumed that the absorption rate of K^+^ would increase in parallel with germination. However, for seeds treated with BONB-KNaCl, we observed a large increase in K^+^ uptake on day 49 (Fig. 3A). This substantial increase suggests that K^+^ ions are more important for growth after germination despite also being necessary for germination. As the experiment progressed, the number of potentially germinating seeds decreased from day 50; therefore, we suspect that the absorption rate of K^+^ continued to decrease. Further investigations should be conducted to establish the interrelationship between K^+^ ions, germination, and growth.

After germination, wasabi seeds need to be cultivated to achieve plant growth. There are two methods of cultivation: direct seeding and vegetative propagation. Because germination of wasabi seeds is notoriously difficult, most researchers and farmers use vegetative propagation despite the caveat of susceptibility to obliteration from pathogens. With potassium-rich BONB treatment, not only would direct seeding be possible, but the potential for genetic diversity would also increase for recalcitrant wasabi seeds even after aging. Moreover, direct seeding would be more advantageous for wasabi seeds because pathogens from the parent plant would not be carried to their seeds, making them pathogen-free. Therefore, combination of potassium-rich BONB treatment and direct seeding would increase biodiversity of wasabi seeds, and this methodology may also be applicable to other recalcitrant species such as avocado, mango, and cocoa.

Until recently, promotion of germination and growth with microbubbles and/or nanobubbles has been proposed to have two mechanisms. One is the removal of growth inhibitors and the other is the facilitation of the supply of gas or nutrients necessary for germination or growth^32^. It is plausible that germination in our experiments was promoted by these two mechanisms by using BNBs. Our study showed that K^+^, Na^+^, and Cl^−^ are necessary for germination. Previous studies have suggested that hydroxyl radicals improved seed germination when strong acid such as hydrochloric acid, and/or H_2_O_2_, was added to the solution^8^, but no such strong acid was added in our study. Other research has also shown that the addition of fusicoccin, a well-known stimulator of germination, increased K^+^ uptake while decreasing the activity of K^+^ out-channels^33^, supporting our proposed model of K^+^ uptake in the facilitation in germination. We believe that it is highly unlikely that radical generation is the only factor in promoting germination. Our results suggest that 2-year-aged recalcitrant wasabi seeds need a supply of ions (K^+^ rather than Ca^2+^) and oxygen (facilitated by BONB in these experiments) for accelerated germination (Fig. 2). Although our study did not investigate the downstream signalling that enables germination, as reported by Tang et al*.*, the Ca^2+^-dependent signalling network may play a role in facilitating an influx of K^+^ ions^34^. As previously reported, ROS from NB solution advanced germination^8^ by stressing the seed to reduce abscisic acid and to release hormones such as gibberellin to initiate germination^35–37^. According to a recent study by Bi et al*.*, hydrogen peroxide (a common ROS) has been reported to serve as a medium for transmitting signals of immune responses according to plant growth^38^. Our hypothetical model (Fig. 3C) shows K^+^ ions attached to BONB being selectively absorbed by the aged recalcitrant seed. Our results showed that K^+^ uptake was slow initially, during dormancy, and then increased significantly as the bud sprouted from the germination process. Selective absorption of certain ions was also reported by Welch and Shuman^39^, which supports our hypothesis.

Our study delves into the mechanism of K^+^ channels and Ca^2+^ pumps in facilitating germination and growth; however, it is possible that other radical ions could be involved in the synergistic mechanism we have proposed. Further studies on genetic diversity through higher germination percentages and better cultivation methods with BONB-KNaCl treatment should be scrutinized. Although this study did not perform stability tests of BONB solutions, BNBs are known to have high stability^12^. Further investigations should be conducted to define the functions, characteristics, and potential values of this technology with other appropriate ions and radicals.

In conclusion, BONB-KNaCl solution may have a direct facilitative effect on germination and growth through ROS formation, radical ions, and constant supply of oxygen. The initial hypothesized process of germination was achieved by stable supply of oxygen from BONB and a salting-out effect from ions. Therefore, use of nanobubble technology to promote germination in high-value plants has the potential to improve seed utilization and reduce germination costs, while increasing the market competitiveness and genetic biodiversity of recalcitrant seeds. Moreover, this novel finding suggests that BONB technology may not only advance the market for recalcitrant seeds and their biological value, but also will have an impact in medical applications such as ultrasound imaging, drug delivery, and cancer therapy^40,41^.

## Methods

### Seed source and seed aging process

Seeds of water-wasabi (*Eutrema japonicum*) were purchased from Wasabi no Monzen (Shizuoka, Japan) in 2019. Nakamura and Sathiyamoorthy^14,15^ described that when wasabi seeds were stored at 5 °C after harvest, the seeds lost their ability to germinate. To replicate this effect and obtain aged recalcitrant seeds, we placed the seeds in plastic bags with silica gel and incubated them at 5 °C for 1.5–2 years. The water content of the wasabi seeds was maintained at 90%.

### Preparation of BONB water

Two types of BONB solutions were prepared and kept for use throughout the experiments from day 1. Milli-Q water used in our study was purchased from WAKO Fujifilm (Osaka, Japan). In the first test, K^+^ and Na^+^ were the main electrolyte cations to test the salting-out effect. In the second test, Na^+^ was the only cation in the initial preparation of the solutions. KCl for K^+^ ions and CaCl_2_ for Ca^2+^ ions (WAKO Fujifilm) were later added to the original BONB-Na solutions. NaOH and HCl solutions (WAKO Fujifilm) were used to adjust the pH level. BONB were prepared from microbubbles as described in our previous report^12^. Microbubbles (diameter < 200 μm) were generated by a decompression-type microbubble generator (A0-1; Dan-Takuma, Yokohama, Japan). Dilute ferrous ion (Fe^2+^) solution was added to stabilize BONB residues. The existence of BONB was confirmed by ESR spectroscopy and AFM (Research Institute of Biomolecule Metrology, Tsukuba, Japan). Figure S1A shows the generated DMPO-OH adduct after adding HCl to the BONB solutions, confirming the production of ROS from the BONB solutions. Figure S1B shows AFM observation of spherical BONB on a mica substrate that had been treated with 3-aminopropyltriethoxysilane. Most of the particles were smaller than 100 nm (Fig. S1B). The BONB were negatively charged because they were deposited on the positively charged substrate by the electrostatic force.

Solutions were characterized by measuring ion concentrations (ion chromatography) and EC (DKK-TOA, Tokyo, Japan), while pH, dissolved oxygen (DO), and temperature were recorded using a DKK-TOA combined pH/DO/temperature meter (see Table 1). All the solutions were formulated with 0.8% and 1.0% ionic concentration with an EC range of 1.64–1.68 mS/cm for the first experiment. EC was measured at 15 °C.

### Seed germination experiments

The germination experiments were performed twice with different experimental conditions. For the first experiment, 216 seeds were divided into four groups for the germination experiments: control (aged recalcitrants) with MQ; aged recalcitrant seeds with MQ and K^+^, Na^+^, and Cl^−^ (MQ-KNaCl); aged recalcitrant seeds with BONB containing K^+^, Na^+^, and Cl^−^ (BONB-KNaCl); and aged recalcitrant seeds with KMnO_4_ solution. A second set of experiments were run with MQ, BONB-KNaCl, BONB-CaNaCl, MQ-KNaCl, and MQ-CaNaCl with 120 seeds.

The aged recalcitrant seeds were placed in a 1.5-L plastic Tupperware container containing 50 mL of treatment solution and were covered with 100% cotton tissue. The solutions were exchanged every 7 days and were sampled before and after. The incubation temperature was maintained at 15 °C.

### Seed cumulative germination analysis

For each treatment group, the number of germinated seeds was counted each day (up to day 65) and the cumulative number of germinated seeds was divided by the total number of seeds for the treatment group. The data were then expressed as percentages.

### Ion chromatography analysis

In the second germination experiment, ion chromatography analysis was conducted once each week during the 65-day trial. Samples (10 mL) were taken from each container with a sterilized pipette tip and immediately filtered through a 0.22-μm PES membrane filter to remove fine suspended particles. Analysis was performed with a Prominence Ion Chromatography Dual System (HIC-20Asuper; Shimadzu, Kyoto, Japan) with a Shim-pack IC-SA3 column for anion analysis and a Shim-pack IC-C4 column for cation analysis (Shimadzu); column temperature, 45 °C; sample volume, 1.8 mL; elution flow rate, 0.8 mL/min; conductivity detection, CDD-10AVP (Shimadzu).

### Statistical analysis

Two-way repeated measures were performed using SPSS Statistics for Windows, Version 25.0 (IBM, Armonk, NY, USA). Other statistical analyses were performed with MATLAB (MathWorks) and Excel (Microsoft). Statistical significance was set at *P* < 0.05.

## Supplementary Information

Supplementary Figure S1.

## Data Availability

The datasets analysed during the current study are available from the corresponding author on reasonable request.
