# Supplementary material for: Accelerated germination of aged recalcitrant seeds by K+-rich bulk oxygen nanobubbles
Source: Sci Rep. 2023 Feb 27;13:3301. doi: 10.1038/s41598-023-30343-2 (PMC9971192; doi:10.1038/s41598-023-30343-2)
Supplement: Supplementary file 1 — Supplementary Figure S1. [file 41598_2023_30343_MOESM1_ESM.docx]

**Electronic Supplementary Information**


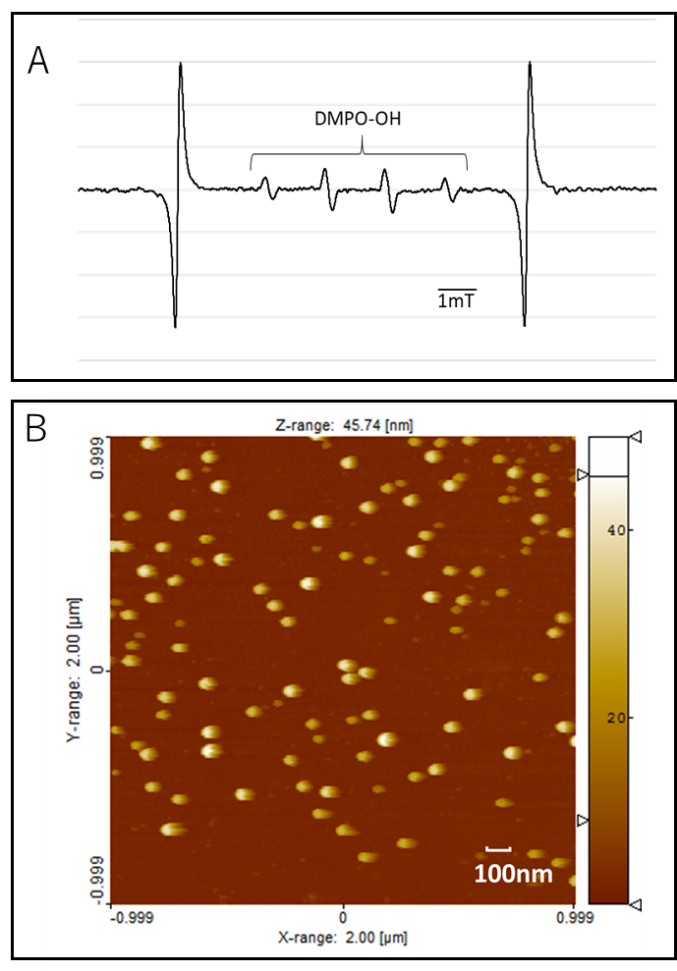


**Fig. S1:** Electron spin resonance (ESR) spectroscopy and atomic force microscopy (AFM) analysis of bulk oxygen nanobubble (BONB) solution. **A** ESR spectrum of DMPO-OH adduct observed using BONB solution. **B** AFM observation of BONB located on mica substrate treated with 3-aminopropyltriethoxysilane.
